# Evidence of learning walks related to scorpion home burrow navigation

**DOI:** 10.1242/jeb.243947

**Published:** 2022-06-23

**Authors:** Douglas D. Gaffin, Maria G. Muñoz, Mariëlle H. Hoefnagels

**Affiliations:** 1 Department of Biology, University of Oklahoma, Norman, OK 73019, USA; 2 Department of Microbiology and Plant Biology, University of Oklahoma, Norman, OK 73019, USA

**Keywords:** Pectines, Peg sensilla, Familiarity, Sensory

## Abstract

The navigation by chemo-textural familiarity hypothesis (NCFH) suggests that scorpions use their midventral pectines to gather chemical and textural information near their burrows and use this information as they subsequently return home. For NCFH to be viable, animals must somehow acquire home-directed ‘tastes’ of the substrate, such as through path integration (PI) and/or learning walks. We conducted laboratory behavioral trials using desert grassland scorpions (*Paruroctonus utahensis*). Animals reliably formed burrows in small mounds of sand we provided in the middle of circular, sand-lined behavioral arenas. We processed overnight infrared video recordings with a MATLAB script that tracked animal movements at 1–2 s intervals. In all, we analyzed the movements of 23 animals, representing nearly 1500 h of video recording. We found that once animals established their home burrows, they immediately made one to several short, looping excursions away from and back to their burrows before walking greater distances. We also observed similar excursions when animals made burrows in level sand in the middle of the arena (i.e. no mound provided). These putative learning walks, together with recently reported PI in scorpions, may provide the crucial home-directed information requisite for NCFH.

## Abstract (precis)

**Highlighted Article:** Evidence that sand scorpions perform looping walks immediately after establishing a burrow and the possible significance of these putative learning walks in terms of scorpion navigation.

## INTRODUCTION

Sand scorpions live in burrows that they dig, and from which they emerge at night to hunt (Polis, 1980; Polis and Farley, 1979). Sand scorpions, especially females, spend most of their time within 1 m of their burrow, though they occasionally venture several meters away (Polis et al., 1985). Questions exist about how they return home. We think scorpions might use a simple view-based navigational process, similar to that proposed for ants and bees, termed ‘navigation by scene familiarity’ (Baddeley et al., 2012; Philippides et al., 2011). However, instead of or in addition to vision, scorpions may be guided by tastes and touches acquired via their mid-ventral pectines (Cloudsley-Thompson, 1955; Wolf, 2017).

Scorpions have both median and lateral eyes, but the paired median eyes seem most conducive to navigation. The median eyes arise from a protuberance on the midline of the dorsal prosoma and gather light from opposing hemispheres of the animal's surroundings. Together, the two eyes have a 360 deg field of view and a 40 deg binocular overlap above the animal (Locket, 2001). The median eyes have high acuity and can detect light as low as 10^6^ irradians (Fleissner and Fleissner, 2001). The median eyes also show sensitivity to polarized light (Locket, 2001; Horváth and Varjú, 2004; Brownell, 2001). Even though the morphology and physiology of scorpion eyes suggest that visual cues such as star patterns and surrounding features and panoramas are accessible to these animals during their night-time forays (Fleissner and Fleissner, 2001), research is lacking on visual navigation in scorpions.

Unlike scorpion vision, the unique scorpion pectines have received considerable attention, in terms of their physiological and morphological characteristics and their potential use in navigation by familiarity. We have developed proof-of-concept models of how scorpions could navigate using ground-based information acquired by their pectines (similar to models of visual navigation in ants; Baddeley et al., 2012). We have termed this process ‘navigation by chemo-textural familiarity’ (Gaffin and Brayfield, 2017; Musaelian and Gaffin, 2020 preprint). Put simply, to get home, the scorpion uses its pectines to detect and move toward tastes and textures it has learned during previous home-bound forays. While the study reported here focuses on the pectines, it is likely the animals integrate chemo-tactile information from their pectines with visual information from their median eyes.

No matter the modality of sensory input, for the navigation by familiarity hypothesis to be viable, two crucial ingredients must be present. First, there must be adequate sensor complexity to match the environment. Second, there must be a way to generate the initial home-bound training paths (Baddeley et al., 2012; Gaffin et al., 2015; Wehner et al., 1996).

Regarding sensor complexity, each pecten has a series of teeth that support thousands of minute peg sensilla (aka ‘pegs’) on their ground-facing surfaces (Ivanov and Balashov, 1979; Foelix and Müller-Vorholt, 1983). Each peg contains a population of chemosensory taste cells (∼10) and at least one mechanosensory neuron that responds when the peg bends (Ivanov and Balashov, 1979; Foelix and Müller-Vorholt, 1983; Gaffin and Brownell, 1997b; Melville, 2000). In all, hundreds of thousands of sensory afferents project from the pectines to the scorpion's central nervous system (Wolf, 2008; Brownell, 2001; Drozd et al., 2020). Based on this complexity, a proof-of-concept model showed that an agent using a downward-facing sensor could navigate various proxies of a simulated environment (Musaelian and Gaffin, 2020 preprint).

**Fig. 1.**
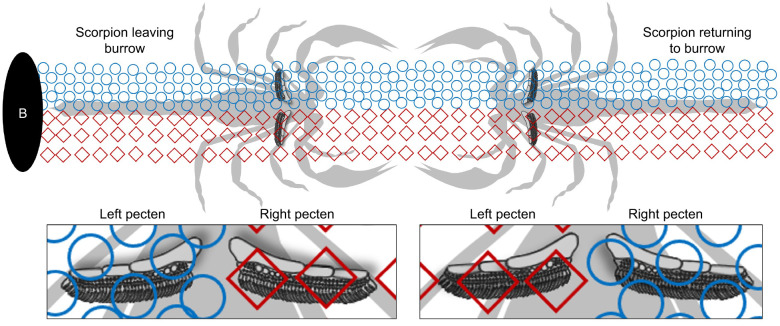
**Conflicting information between outbound versus inbound paths.** The chemicals and textures the pectines experience on the journey leading away from the burrow (B) depart 180 deg from what they experience on the return trip to the burrow.

Sensory complexity is therefore adequate; what about the generation of home-bound training paths? The glances and tastes a scorpion experiences while leaving its nest or burrow depart 180 deg from those that lead home (Fig. 1). How does the animal know its way home after venturing out for the first time? Innate behaviors such as path integration (PI) and learning walks may provide the answer. In PI, the distance and direction of each outbound leg is integrated to compute an approximate homebound vector (Wehner, 1992; Papi, 1992). PI is well documented for many animals, but the studies of desert ants are the most extensive (Collett, 2019; Wehner, 1992; Wehner and Srinivasan, 1981; Wehner et al., 1996, 2004, 2006; Wolf, 2011; Wittlinger et al., 2006, 2007; Wittlinger and Wolf, 2013; Heinze et al., 2018; Srinivasan, 2015). PI has also been described for some groups of spiders (Ortega-Escobar, 2002, 2006; Ortega-Escobar and Ruiz, 2014, 2017; Görner and Claas, 1985; Moller and Görner, 1994; Nørgaard, 2005; Seyfarth and Barth, 1972; Seyfarth et al., 1982), and a recent study showed evidence of PI in the lesser Asian scorpion, *Mesobuthus eupeus* (Prévost and Stemme, 2020).

In addition, learning walks are innate behavioral patterns thought to further help the animal gain goal-directed stimuli (Deeti and Cheng, 2021; Müller and Wehner, 1988, 2010; Ronacher, 2008; Wehner et al., 2004; Fleischmann et al., 2016, 2017, 2018; Zeil et al., 1996, 2014). As with PI, learning walks are well described for navigating ants (Jayatilaka et al., 2018; Zeil and Fleischmann, 2019) but have never been documented for scorpions or any other arachnid (although the zigzag outbound paths of the wandering Namib spider, *Leucorchestris arenicola*, are strongly suggestive; Nørgaard et al., 2012; Gaffin and Curry, 2020).

In this study, we made long-term video recordings of sand scorpions as they produced burrows in the middle of laboratory arenas. We show that the animals make consistent, repeated looping paths immediately after their first burrow-digging behavior and that these paths have similar characteristics to learning walks in ants.

## MATERIALS AND METHODS

### Animals, collection details and maintenance

Desert grassland scorpions, *Paruroctonous utahensis* (Williams 1968), were collected from the Walking Sands dune area about 6 km SE of the University of New Mexico Sevilleta Field Station, NM, USA. We used UV lights to find animals on three nights during periods of new moon in August, September and October 2020. Only animals judged to be adults were collected. Fig. S1 shows the collection locations and the mixture of males and females from the three collection nights. We collected many more males than females in the August and September collections, but many more females than males in the October collection. The animals were transported and housed individually at the Station in small rectangular food storage containers with air holes drilled in the lids and ∼50 ml of sand collected from the animals' habitat as a substrate. The animals were exposed to a 14 h:10 h light:dark cycle (lights on at 06:00 h, off at 20:00 h) using indirect light from two white 60 W equivalent LED bulbs housed in work lights (Bayco clamp light, 21.6 cm) placed ∼50 cm from the animals and plugged into a timer switch. The room temperature was maintained at about 22°C. After 45 days, we moved all animals to a room in the laboratory building on the UNM Sevilleta campus where the animals were exposed to natural light that streamed through the large NE facing picture windows and the temperature was kept at 20–21°C with a relative humidity of 16–20%. A voucher specimen was given to the Sam Noble Oklahoma Museum of Natural History at the University of Oklahoma in Norman, OK, USA.

### Encouraging burrow formation

We noticed some patterns of digging activity in pilot studies of animals atop native sand in circular arenas (for an example, see Fig. S2). We ran several additional pilot studies to determine which conditions were most conducive to the scorpions digging and occupying burrows. These included tests of various substrates (Fig. S3), mound sizes and sand moisture content (Fig. S4), and the timing of burrow occupation relative to daylight (Fig. S5).

### Behavioral apparatus and video recording

**Fig. 2.**
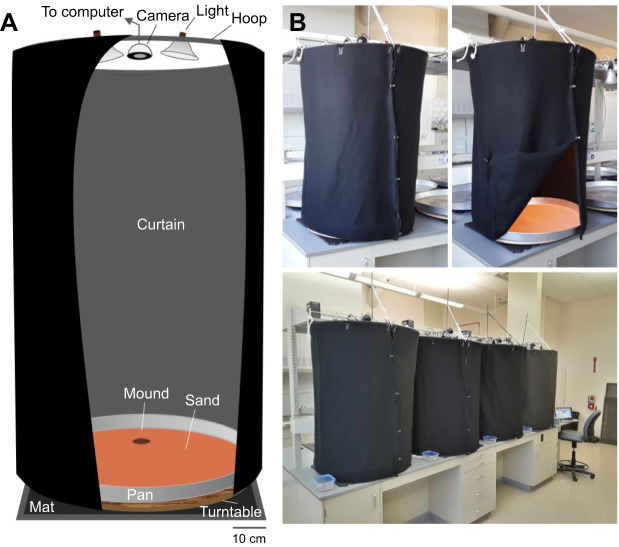
**Behavioral set-up for long-term recordings.** (A) Each arena was composed of an aluminium heater drain pan atop a turntable and a rubber mat. Sand was added to the pan and a mound was formed in a pre-defined location. A curtain was cut from black, light-blocking material and suspended from a hula hoop attached to a supporting frame. Two timer-controlled lights and an infrared camera connected to a laptop computer were also attached to the frame. (B) Photos of an arena with curtain closed (left) and open (right) and the four arenas (below) arrayed along the lab counter with the laptop computer at the end.

We built four identical behavioral set-ups (Fig. 2) in the UNM Sevilleta Field Station lab building (inspired by Vinnedge and Gaffin, 2015). Each arena consisted of an aluminium water heater drain pan (Camco, product no. 20860; 76.6 cm base diameter, 7.6 cm height) sitting atop a turntable (formed from a 70 cm diameter×1.9 cm thick plywood disk attached to a 30.5 cm diameter Richelieu swivel plate with 454 kg capacity) to allow 360 deg rotation. A rubber mat (Ottomanson multi-purpose 61×61 cm exercise tile mat) was placed beneath each arena to dampen room vibrations. About 1250 ml of screened native sand was spread in a thin layer across the bottom of each arena. We then added ∼250 ml of native sand through a small funnel to form a mound in the middle of the arena. We then misted the mound from above with 20 squirts of water (∼15 ml). To minimize the role of vision, light- blocking curtains were secured to hula hoops (Ice Hoop, Kess Co.; 86 cm diameter) with large binder clips and draped around each arena. Two work lights (Bayco clamp light, 21.6 cm) equipped with broad-spectrum bulbs (Duracell Ultra 75 W equivalent daylight) were positioned 110 cm above each arena. The lights were controlled by a timer set to a 14 h:10 h light:dark cycle (on at 05:30 h, off at 19:30 h). We used infrared cameras (ELP 1 megapixel Day Night Vision) to track the animals; scorpions do not appear to perceive infrared light (Fleissner and Fleissner, 2001). The cameras were mounted 110 cm above the center of each arena and connected via USB to two laptop computers (two cameras per laptop; Apple MacBooks). A MATLAB script was written to toggle between the cameras and acquire 200×200 pixel frames at a user-defined interval. The frames were stored in a MATLAB structure array for subsequent analysis.

**Fig. 3.**
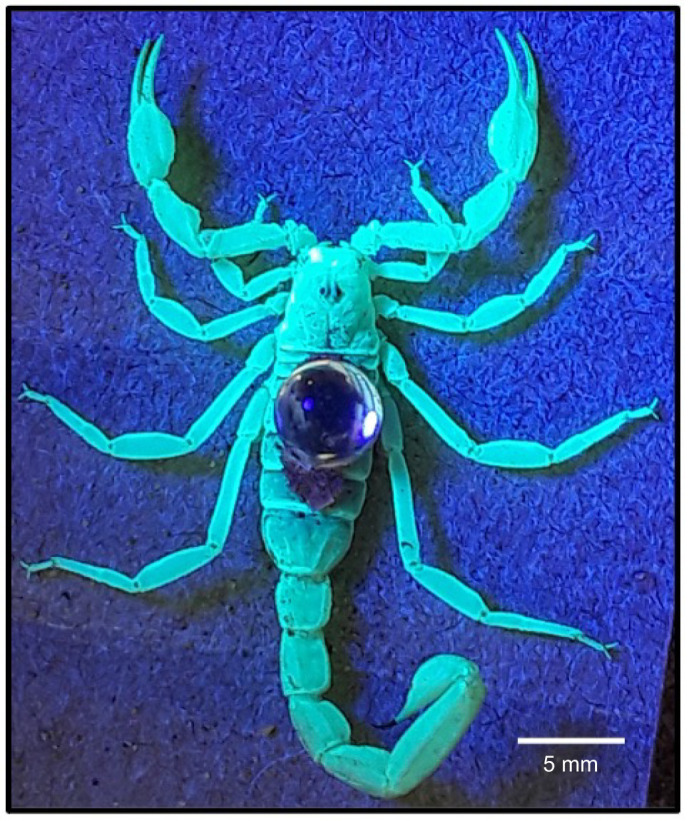
**Animal with attached crystal.** A *Paruroctonus utahensis* female photographed under UV light with a 5 mm round cab crystal affixed via double-sided tape to its dorsal mesosoma.

To aid in video tracking, we used double-sided tape to affix a small crystal (5 mm round cab crystal; Acrylic Gems) on the dorsal mesosoma of each animal before releasing them into the behavioral arenas (Fig. 3). To secure the crystal, we first placed an animal in a rectangular plastic container (30×17 cm). We then placed a square plastic sheet (8.5×8.5 cm) that had a 6 mm hole cut close to one of its corners over the animal such that the hole was over the mesosoma with the remainder of the sheet covering the rest of the animal's body. This system calmed and secured the animal and allowed the crystal to be easily applied through the hole to the animal's back without the danger of being stung. The crystals reflected infrared light from all angles and from all animal positions within the arenas, so the plotting accuracy in MATLAB was greater than 99%. Smaller 3 mm crystals proved less effective, given the camera's resolution and distance from the arena floor.

Before each recording, we created a mound either in the center of an arena or offset from the center in various positions. The video monitoring system was then set to record for a given length of time. We set our recording times to focus mainly on the animals' most active periods while also managing our digital storage capacity. As such, some gaps in the recordings are apparent during daytime hours. Finally, a crystal-equipped animal was introduced near the wall of its designated arena and the curtains were completely closed around the front of the set-up using binder clips.

### Inducing learning walks without mounds

We also induced scorpions to occupy burrows in the center of the arenas without pre-made mounds. To do this, we added a thicker layer of sand (3000 ml) to the arena and placed a plastic ring (30 cm diameter×12.5 cm tall) in the middle. In the center of the plastic ring, we partially buried a small paper slip (formed by removing the base of a Dixie 3-ounce bath cup). The slip was 4 cm in diameter with a 4 mm tall rim that had a quarter of its circumference removed; the rim side was placed downward. We misted over the top of the slip with 5 squirts (∼4 ml) of water to provide additional structural support to induce the scorpion to dig within this smaller arena. We then placed a scorpion in the center ring in the late afternoon and used video recording and MATLAB to track the animal's movements for 18–22 h. The plastic ring was removed the following afternoon if the scorpion was found inside or near the burrow. If the scorpion failed to dig into the region of the paper slip, the smaller ring was left in place, the region was misted with three additional squirts of water, and the animal was given an additional night to burrow. After the plastic ring was removed, we continued recordings to track the animal's movements throughout the large arena for another night.

### Analysis

We wrote various MATLAB scripts to analyze our behavioral data. We used a frame-by-frame subtraction method coupled with centroid plotting to automatically track the *X*–*Y* coordinates of the scorpion locations during our videos. We then used the Pythagorean theorem to calculate the distance walked and used the video frame capture rate to determine velocity. We also made time-lapse videos that plotted the current animal position along with the three previous positions to create a stardust effect, which efficiently revealed instances of the animal's initial burrowing. Once the initial digging was identified, we then hand plotted (for increased accuracy) the animal's subsequent movements until we were confident that the animal had resumed its exploratory behavior or remained in the burrow for a prolonged period.

## RESULTS

### Activity plots

**Fig. 4.**
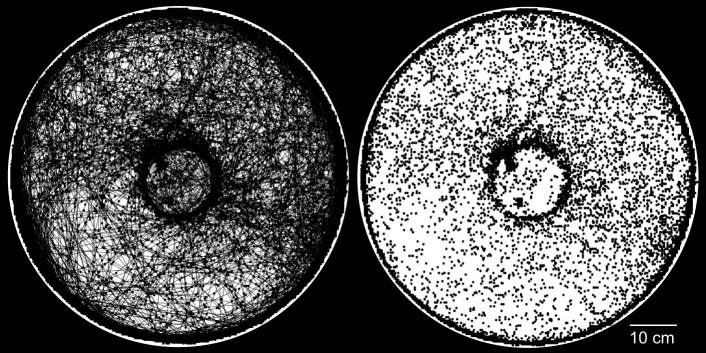
**Long-term activity plot.** Example of an all-night video with the animal's position plotted every second via a MATLAB script. The animal's paths are shown by connecting the points with line segments in the left plot; the segments are excluded in the right plot.

In all, we tracked 23 different animals (14 males, 9 females), some for multiple evenings, for a total of nearly 1500 h of video. During our trials, the animals spent most of their time walking along the walls of the arena but also made many forays across the arena's interior. All-night plots of animal movements (Fig. 4) showed a lot of activity, including concentrated movements around the central mound.

### Burrow formation

**Fig. 5.**
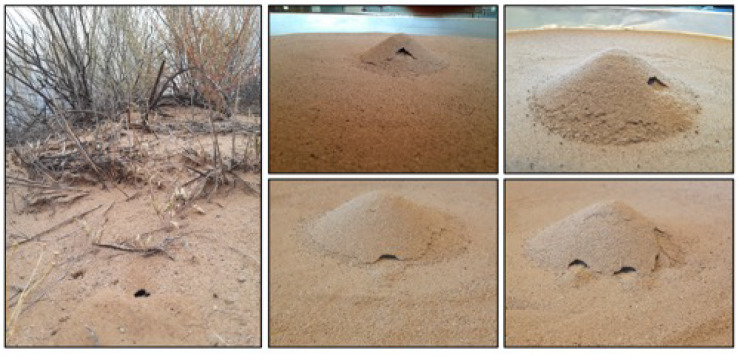
**Scorpion burrows in nature and in the lab.** The photo on the left is an example of a scorpion burrow next to one of the field station's trails. The four photos on the right are examples of burrows we saw in our trials in the lab.

As in our pilot studies (see Figs S3–S5), the animals in these trials readily dug burrows in the central mounds. Most of the initial digging occurred toward the end of the dark period or soon after the lights were turned on. A sample of some of the burrows we observed is shown in Fig. 5, along with an example of a natural scorpion burrow filmed on the wildlife refuge. A short video clip of a scorpion digging its burrow in the lab is also provided (Movie 1).

### Activity patterns

**Fig. 6.**
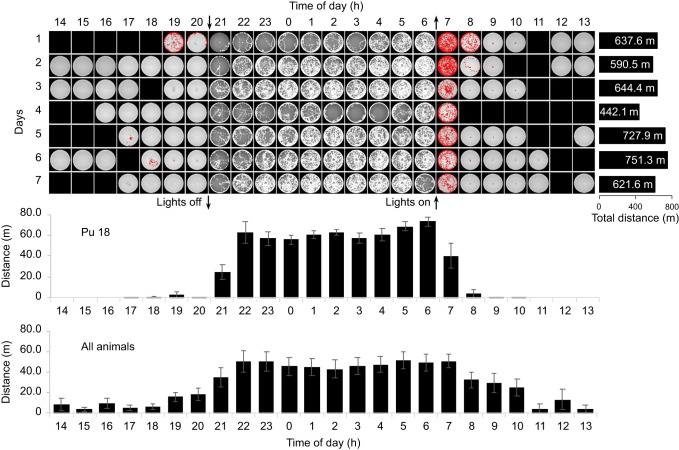
**Animal activity patterns.** Top: plots for a single animal over 7 consecutive days. The arrows indicate when the lights were turned off (down arrow) and on (up arrow). The blank squares reflect periods when the video recording was paused. The distance walked each night is shown in the bars on the right. Middle: histogram (Pu 18) showing the mean (±s.d.) distance walked by hour for the animal depicted in the top plots. Bottom: summary histogram of all the animals we tested (23 animals, ∼1500 h of video sampled every 2 s).

Fig. 6 (top) shows activity plots by hour for an animal we monitored for 7 consecutive days. Over the 7 days, the animal walked 4415 m for an average of 631 m per night. Tracking this animal's average distance walked by hour of the day showed a consistent pattern of behavior (Fig. 6, middle) with the highest activity soon after the arena lights were turned off in the evening and just before or just after the lights were turned on in the morning. This pattern was also evident when the activities of all animals were pooled (Fig. 6, bottom).

### Signs of learning walks

**Fig. 7.**
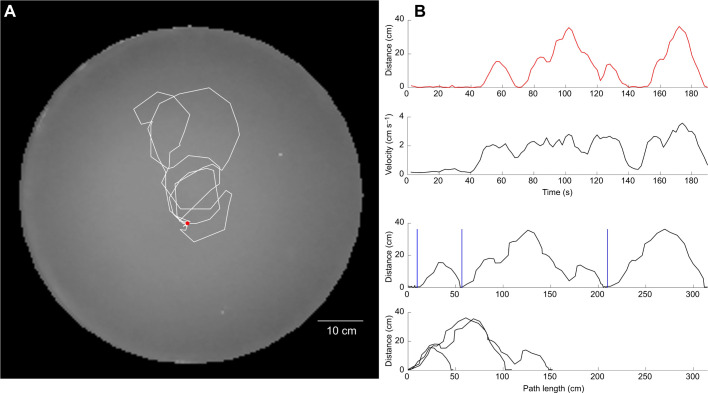
**Sample learning walks and processing.** (A) Approximately 200 s of an animal's initial learning walk is plotted (red dot, burrow). (B) We processed the walks by first plotting distance from the burrow by time (first graph) and the velocity of the animal by time (second graph). We then transformed the data to linear distance from the burrow against cumulative path length (third graph) and sliced out the walks based on each return to the burrow (vertical blue lines), then superimposed the walks (fourth graph).

We were interested in the locomotory movements the scorpions made immediately after their first digging behavior in the central mound. We identified these using time-lapse video and plotted the animal's movements away from and back to the initial digging position. An example of a typical learning walk following initial burrowing is shown in Fig. 7; a video of this walk is also provided (Movie 2). Fig. 7 also shows how we processed video showing the looping excursions. We hand-marked the position of the burrow and used the Pythagorean theorem to plot the distance of the animal from the burrow over time (Fig. 7B, first graph). We also plotted the animal's instantaneous velocity by time during the walk (Fig. 7B, second graph). Next, we plotted the distance from the burrow against the cumulative path length (Fig. 7B, third graph) and marked each return to the burrow. Finally, we superimposed each of these individual loops by plotting the start of each at the origin (Fig. 7B, fourth graph).

**Fig. 8.**
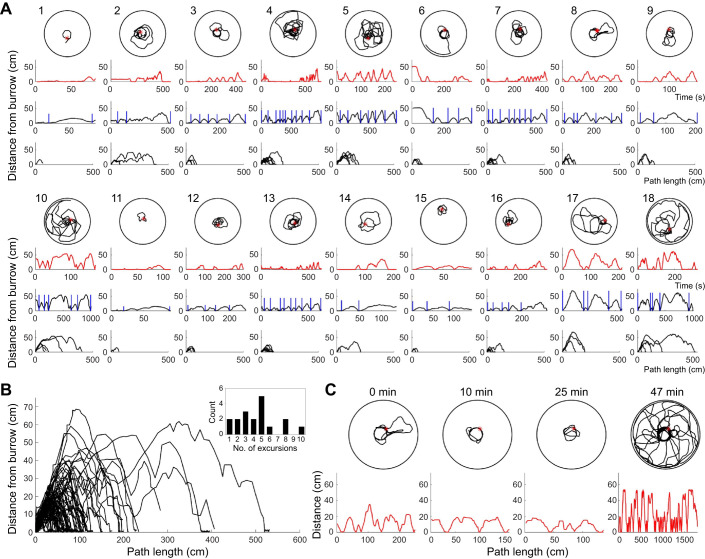
**Learning walks.** (A) All 18 sets of initial learning walks observed in the study are plotted and processed as described in Fig. 7. (B) All 80 looping excursions from the 18 animals are superimposed. The inset shows a frequency distribution of the number of animals with each number of excursions. The number of excursions ranged from 1 to 10 and averaged 4.4 per animal. (C) The animals often made subsequent looping excursions later in the recordings. This example shows additional excursions 10, 25 and 47 min after the first set of walks.

Of the 23 animals we monitored, 18 showed looping walking behavior immediately after the first signs of burrow digging. For the other 5 animals, the video resolution either did not allow accurate detection of digging behavior or burrow formation happened outside the period of video monitoring. Fig. 8A shows all the initial learning walks that we encountered for these 18 animals along with the processing described in Fig. 7. In all, 80 looping excursions away from and back to the burrow were identified for all the animals and these are superimposed in Fig. 8B. The number of loops observed per animal varied from 1 to 10 and averaged 4.4±2.5 (mean±s.e.m.). The average duration of the initial learning walks was 348.9±47.9 s and the average distance covered was 505.6±74.6 cm. We determined the average velocity of each animal's initial learning walk by dividing the distance covered by the duration of the walk. The average velocity of these walks was 1.7±1.4 cm s^−1^.

The focus of this study was on capturing the first occurrence of putative learning walk behavior immediately after the initial signs of burrow digging. However, the animals displayed many subsequent looping routes later in the videos. We judged these to be learning walks instead of foraging trips because they were continuous (i.e. lacked prolonged pauses), included repeated returns to the burrow, and did not involve extended bouts of wall-walking. Some of these routes were elaborate and encompassed all parts of the arena. One such example is shown in Fig. 8C, where bouts of looping excursions occurred 10, 25 and 47 min after the initial set.

### Learning walks without a mound

**Fig. 9.**
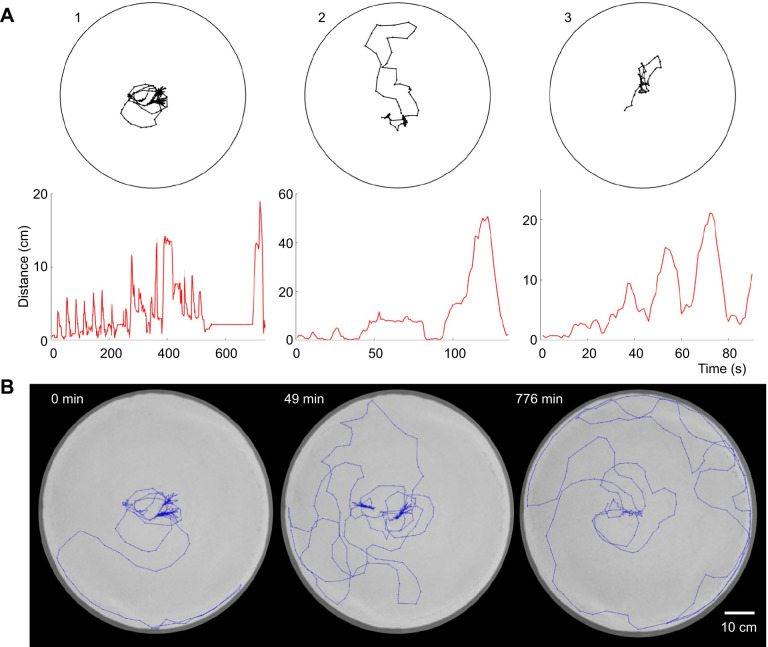
**Learning walks without a mound.** (A) Examples of the initial set of looping excursions for three animals that were induced to burrow in level sand in the middle of the arena. (B) Example of multiple bouts of looping excursions for animal 1 in A. The initial set (time 0) is shown on the left; the other two sets were detected at 49 and 776 min after the initial bout. The bouts were 21, 19 and 11 min long, respectively.

We tried to reduce the possible visual or tactile influence of the sand mound by inducing animals to adopt burrows in level sand in the middle of the arena. We first confined the animals to a smaller ring (30 cm diameter) in the middle of the large arena, along with a partially buried paper slip to encourage burrowing. After we were convinced that the animal had occupied the burrow, we lifted the ring to allow the scorpion access to the rest of the large arena. Fig. 9A shows three examples of the first set of looping excursions that animals made after their first return to the burrow in the middle of the arena. Fig. 9B shows an example of subsequent bouts that occurred later in the recording.

### Arena rotation

**Fig. 10.**
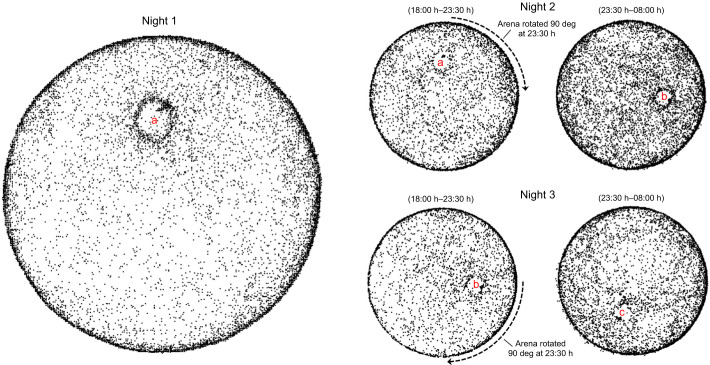
**Animal behavior after arena rotation.** Three nights of activity are shown. During night 1, the arena was maintained in its original orientation. Midway through night 2, the arena was rotated clockwise 90 deg. The position of the burrow can be detected in the night 2 plots before (position ‘a’) and after (position ‘b’) the rotation. Midway through night 3, the arena was again rotated clockwise 90 deg. The position of the burrow can be detected in the night 3 plots before (position ‘b’) and after (position ‘c’) the rotation.

We tried rotating four arenas midway through two successive evenings when the animals were away from their burrows to see if the change in burrow position relative to the room and surrounding curtain affected burrow relocation. Of the four animals, two either stayed in their burrow or did not move enough prior to the rotation to produce a detectable pattern in their movements. The video resolution for the third animal was too poor to accurately resolve its paths. However, the fourth animal's video was good and contained enough movement points to show patterns before and after the rotation on each of the successive nights. This animal's movements for the 3 nights are shown in Fig. 10. The animal repeatedly returned to the rotated burrow (instead of the position prior to rotation).

## DISCUSSION

Our findings are clear. Essentially all animals that made their own burrows in the middle of our laboratory arenas executed looping walks immediately after their first signs of digging. We found similar looping excursions whether we induced the animals to burrow in a small sand mound or in level sand in the middle of the arenas. This is the first report of learning walks in scorpions.

### Potential role of learning walks in familiarity navigation

Learning walks are consistent with navigation by both visual and chemo-textural familiarity. In line with familiarity navigation, the putative learning walks could be an innate behavior that allows scorpions to acquire home-directed views, tastes and touches near their burrow for subsequent retracing (Baddeley et al., 2012; Gaffin and Brayfield, 2017; Musaelian and Gaffin, 2020 preprint). This idea is similar to that proposed for familiarity navigation in desert ants (Baddeley et al., 2011, 2012). In addition to panoramic information gathered by the eyes, the scorpion pectines could act as local sensors that acquire matrices of chemo-textural information from the substrate beneath the animal. This local sensor approach was used in a computer simulation that used straight-down views of Earth satellite images to navigate (Gaffin et al., 2015) and has been applied to simulations of scorpion navigation (Gaffin and Brayfield, 2017; Musaelian and Gaffin, 2020 preprint).

In hymenopterans, learning walks and learning flights appear to help the animals learn home-related features of the landscape (Degen et al., 2016; Collett and Zeil, 2018). These walks or flights, if directed in various directions from the hive or nest, also keep an animal from overshooting its home when following a longer home-bound vector (as generated by PI). This is because the scenes, tastes and touches beyond the nest would be unfamiliar unless there was a way to acquire a repertoire of home-directed scenes which bend back to the starting point. Indeed, the addition of artificial learning walks to a computer simulation improved the homing accuracy of artificial agents navigating by scene familiarity (Baddeley et al., 2012).

A few of our observations are consistent with the navigation by familiarity hypothesis. First, the animal observed in the rotated arena experiment (Fig. 10) returned faithfully to its burrow instead of the burrow's position prior to rotation. We were not surprised that the animal found its burrow as the burrow's position and substrate did not change relative to the animal's position (even though the surrounding visual information did change). While this behavior is supportive of the chemo-textural familiarity idea, it can also be explained by PI. Second, we also found some interesting movement patterns during a subsequent assessment of inbound and outbound paths relative to burrow location (Fig. S6). We digitally placed a rectangle around the position of the animal's burrow after movement coordinates had been gathered for an entire evening. We then used MATLAB to plot the 20 s of movement prior to the animal entering the rectangle (‘Inbound’ paths) and the 20 s of movement after the animal exited the rectangle (‘Outbound’ paths). Interestingly, the animals seemed to follow more consistent and concentrated inbound paths compared with their more dispersed outbound paths. These movement patterns suggest that previously learned features (visual, chemical, textural or other) may guide animals along consistent home-directed routes.

### Sensor complexity of the pectines

In the Introduction, we noted that adequate sensor and environmental complexity is necessary for animals or agents navigating via familiarity to avoid being confused by similar scenes, tastes or textures in multiple locations. This trade-off has been examined in various vision-based simulations (Gaffin et al., 2015; Gaffin and Brayfield, 2016) and in a navigation simulation modeled on scorpion pectines (Musaelian and Gaffin, 2020 preprint). Our estimates of the pattern detection capacity of scorpion pectines are informed by electrophysiological studies showing that peg sensilla responded similarly to a variety of chemicals presented to the pore at the peg tip (Knowlton and Gaffin, 2009, 2010, 2011a,b; Gaffin and Walvoord, 2004). Based on these data, it has been estimated that the pectines can conservatively detect from 10^12^ to 10^40^ different patterns (Gaffin and Brayfield, 2017). Further, neurons in peg sensilla interact synaptically (Gaffin and Brownell, 1997a; Foelix and Müller-Vorholt, 1983; Melville, 2000; Gaffin, 2002), which appears to reduce sensory adaptation through a local feedback loop and may improve information fidelity for navigation (Gaffin and Shakir, 2021).

Quantifying the chemo-textural complexity of the scorpion's sand substrate, however, is difficult. Proxies of the textural information available on the surface of a fine sand substrate (and at dimensions germane to the packing densities of the peg sensilla matrices) have been generated by photographing multiple images of sand through a dissecting microscope while projecting light from the side to produce pronounced shadows (Gaffin and Brayfield, 2017; Musaelian and Gaffin, 2020 preprint). Knowing the nature of the chemical milieu that occurs naturally on sand grains is still more challenging. Studies of scorpion responses to pheromone deposits suggest the chemicals may stably adhere to the sand grains and remain viable for scorpion sensory detection for days to weeks (Gaffin and Brownell, 1992; Taylor et al., 2012). It seems safe to suggest that decaying organic matter, animal deposits and numerous other processes leave hundreds of residual chemicals on the sand in varying concentrations, creating enormous chemical complexity. Simply put, the peg matrices and substrate appear suitably matched in complexity.

### Other interpretations for learning walks

While the looping paths that we observed could be learning walks for gathering homebound information for familiarity navigation, we cannot rule out other interpretations. For example, female *P. utahensis* are known to release ground-based pheromones to attract males during the mating season (Taylor et al., 2012). As such, it seems possible that the animals might be releasing their own chemical cues during the loops to generate a burrow-centered gradient of markers that they could use to orient back to their burrow after future excursions. It is also possible that the learning walks serve to gather information about what is around a newly established burrow (such as conspecifics, prey, predators). Furthermore, sand scorpions hunt by detecting vibrations (Brownell, 1977) and can be drawn meters away from their burrow (Polis et al., 1985; Gaffin, 2011), which makes them more vulnerable to predators (e.g. owls, grasshopper mice, etc.). It is therefore adaptive for scorpions to return quickly and accurately to their burrow shelter. One possibility is that the learning walks simply facilitate a quick retreat to safety and play no role in subsequent navigation.

### PI as the mechanism of learning walks

There is an intimate relationship between learning walks and PI, and it is likely that aspects of PI underlie the generation of locomotory movements that bring the animal back to its initial digging point. The information required for PI can be disaggregated into directional and distance components. Directional cues are often deduced by updating the animal's outbound bearing as compared with a reliable external reference, such as polarized light patterns or the Earth's geomagnetic field (Wehner, 1992; Papi, 1992; Gaffin and Curry, 2020). Directional information can also be gleaned from differential activation of joint-associated lyriform organs to monitor turns during sinuous outbound journeys (Seyfarth and Barth, 1972; Seyfarth et al., 1982). Distance estimates are also necessary for successful PI and this information can be assessed by monitoring the animal's own movement with mechanisms such as counting footsteps (Wittlinger et al., 2007; Wolf, 2011) or monitoring optic flow across the animal's eyes (Wolf, 2011). It will be interesting to assess the mechanisms that underpin the scorpion's distance and directional computations during learning walks by selectively ablating or covering specific sensory organs, such as leg slit sensilla (including basitarsal compound slit sensilla; Brownell, 1977; Brownell and Farley, 1979), the median and lateral eyes, and the pectines.

### Future studies

Many additional studies are needed to build on the results presented in this study. Most of our attention has been on the pectines, but we cannot ignore the likely contribution of vision in any of our arguments. The current study does not provide explicit evidence that scorpions are using texture or chemical information to find their way back home. Future studies of navigation need to test animals with their eyes and pectines selectively covered or ablated to see whether homing ability is compromised. While we ran our experiments under infrared cameras and attempted to exclude as much extraneous light as possible, scorpion eyes are sensitive to starlight levels of light (Fleissner and Fleissner, 2001). It is therefore crucial to repeat these tests using animals whose eyes have been thoroughly blocked with blindfolds. The arena lights should also be smoothly dimmed and brightened to simulate natural sunset and sunrise conditions. While removing the pectines could be harmful to the animal, it might be possible to reversibly cover the pectines with tubing or tape to assess the use of these organs relative to chemo-tactile information. Other experiments should consider disrupting the sand around the burrow after bouts of walks have occurred to see whether looping behavior intensifies relative to baseline levels without disruption. In addition, disruption of the sand while the animal is away from its burrow would be useful for assessing the use of home-directed substrate information. In future incarnations of the rotated arena experiment, the animal should be lifted prior to arena rotation and repositioned in a new position relative to the burrow. If chemo-textural familiarity information is salient, we would expect the animal to be drawn to the new position of the rotated burrow instead of the previous burrow location. Tests also need to systematically alter the rotation of the arena relative to the curtain and the laboratory to control for visual and geocentric cues. Displacement studies in which animals familiar with one arena are captured while away from their burrow and transferred to a novel arena would also be useful for assessing the use of PI in generating a home-directed vector (see Ortega-Escobar, 2002, for one such model). Finally, we think it would be interesting to look for signs of learning walks in other long-range navigating arachnids, such as amblypygids, that have substantial chemosensory and mechanosensory attributes (Hebets, 2002; Hebets et al., 2014).

## Supplementary Material

Supplementary information
