# Supplementary material for: Evidence of learning walks related to scorpion home burrow navigation
Source: J Exp Biol. 2022 Jun 23;225(12):jeb243947. doi: 10.1242/jeb.243947 (PMC9250797; doi:10.1242/jeb.243947)
Supplement: Supplementary information [file jexbio-225-243947-s1.pdf]

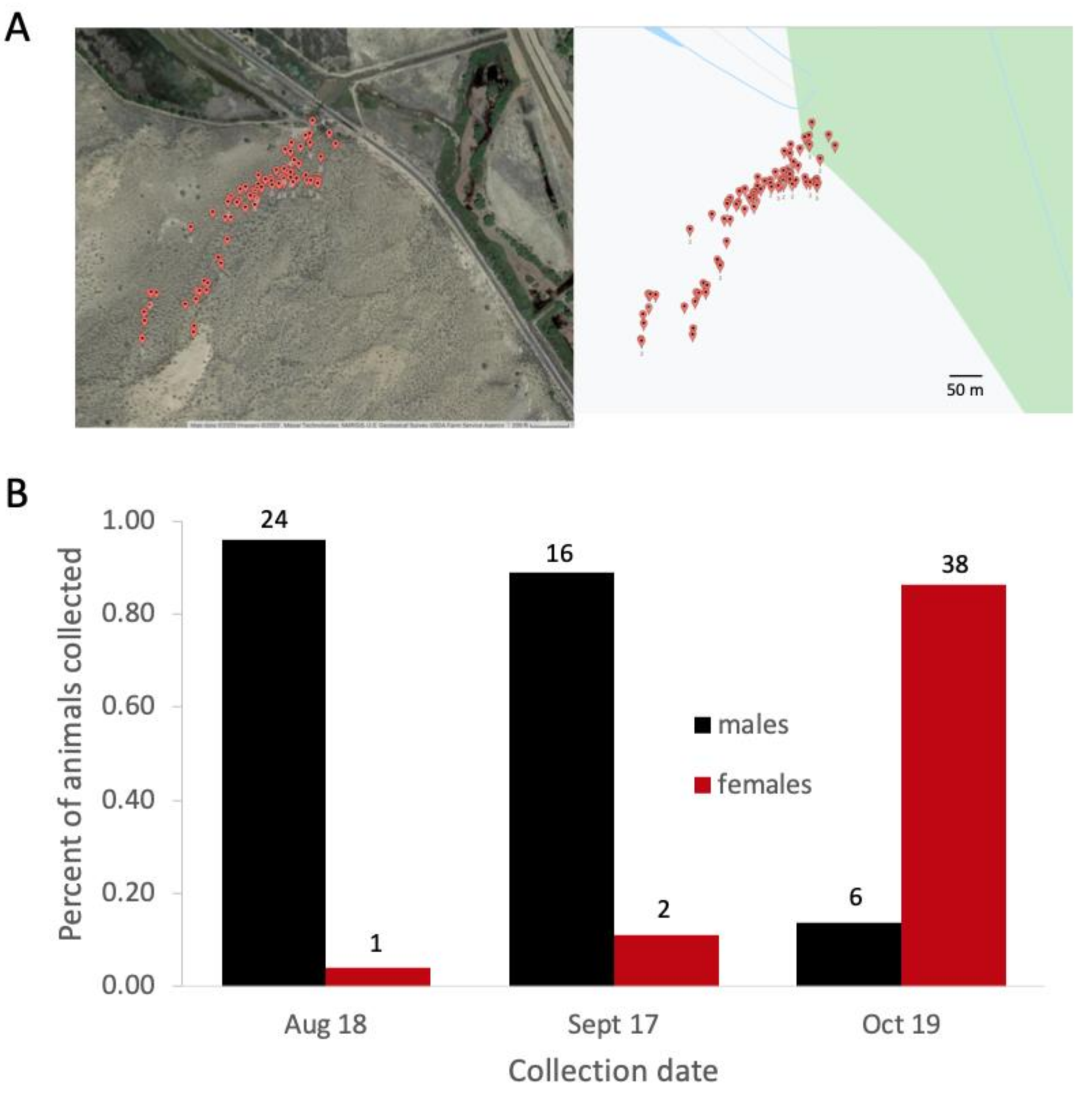

Fig. S1. Collection details. (A) Markers indicate the locations of animals obtained during the three nights of collection from sandy areas at the junction of the Sevilleta refuge (green shading) and the La Joya Management Area (white) in 2020. Upper most marker is the parking spot at 34.302072 (lat), -106.85465 (long). (B) Mix of males and females obtained on the three collection nights.

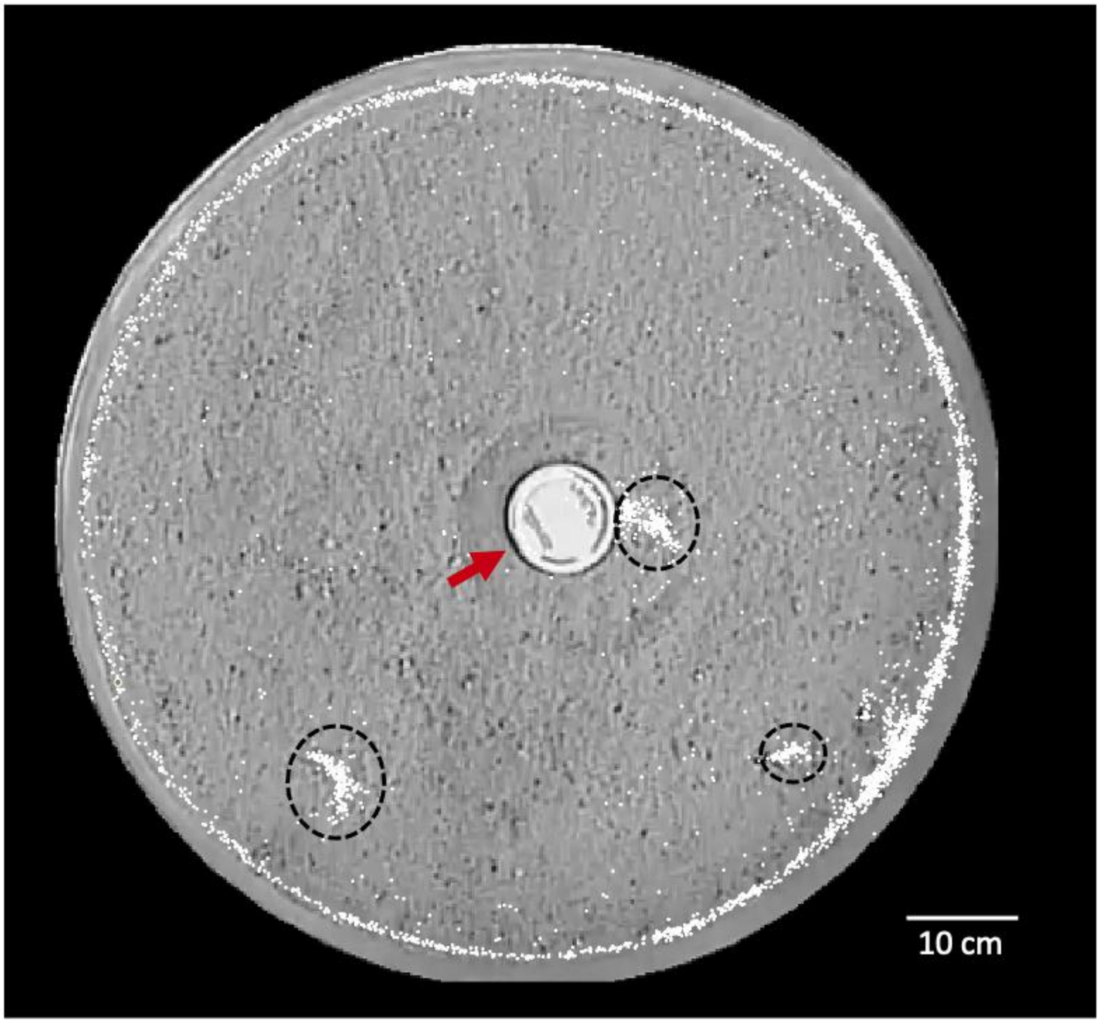

Fig. S2. Initial observations of long-term scorpion activity. In a pilot study, a male *P. utahensis* was placed in a 76 cm diameter water heater drain pan lined to a depth of about 3 cm with sand from the animal's native habitat. A canning jar lid with a small section of the rim removed was placed in the center of the arena to serve as a proxy for a burrow. The arena was placed outside the casita at Sevilleta and monitored through the night from above with a tripod-supported IR camera. The animal's location was plotted every second for several hours. The plot shows the animal did not use the lid but returned frequently to a spot on the opposite side of the lid's opening that was conducive to digging – where the sand appeared a little deeper and stabilized by some moisture (some water was sprinkled over the arena prior to introducing the animal). The animal also focused on two other spots in the arena (besides the typical wall-walking behavior). This observation suggested the need to allow the animal to dig its own burrow and led to a series of tests to determine what conditions were conducive to animals digging their burrow in places useful to our assay. [The red arrow points to the opening in the metal lid refuge; the three dashed black circles indicate locations where the animal returned repeatedly to dig.]

Journal of Experimental Biology • Supplementary information

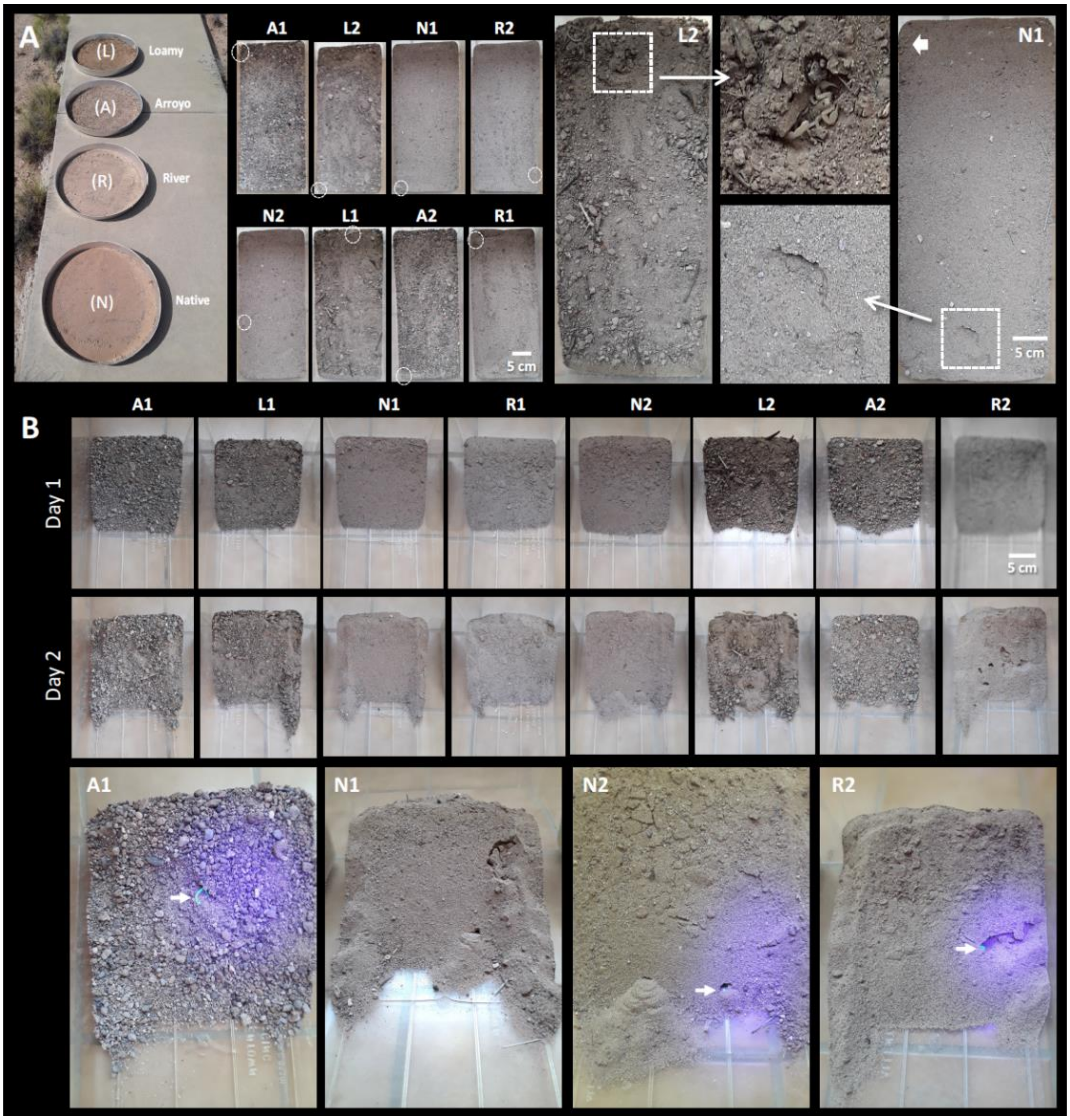

Fig S3. Animal behavior atop various substrates. **A** Pans containing the four tested substrates (left) were placed in the sun to dry. Individual *P. utahensis* males were monitored in rectangular plastic containers (center) each holding ~1.4 L of leveled substrate. Replicates of four substrates (A=arroyo, L=loamy, N=native, R=river) were placed in eight arenas, which were placed in front of a NW facing window in a scrambled order. The animals are indicated by dashed circles in these snapshots. The positions of the animals and evidence of burrow formation were checked hourly for eight hours beginning at 12:45. After about three hours (right), an animal in the loamy substrate dug and settled into an impression around a small twig, and an animal in one of the native substrates had dug a well-formed burrow but was found in one of the corners of the arena (white arrow). **B** Slope study. The top row shows the substrates sloped against one of the end walls of each box (A=arroyo, L=loamy, N=native, R=river). We also misted the sloped substrates with 40 squirts of water (~30 ml) before placing the same animals as before into their respective arenas. The second row shows the same substrates after two days of animal activity. Distinct burrows were formed in four of the boxes: one of the arroyo sands, both native substrates, and one of the river sands (bottom row). The animals can be detected in their burrows under UV light in A1, N2, and R2 (white arrows). The two animals in the native substrate built well-formed burrows near the base and center of the slope. The river sand animals tried, but the walls of their burrows collapsed.

Journal of Experimental Biology • Supplementary information

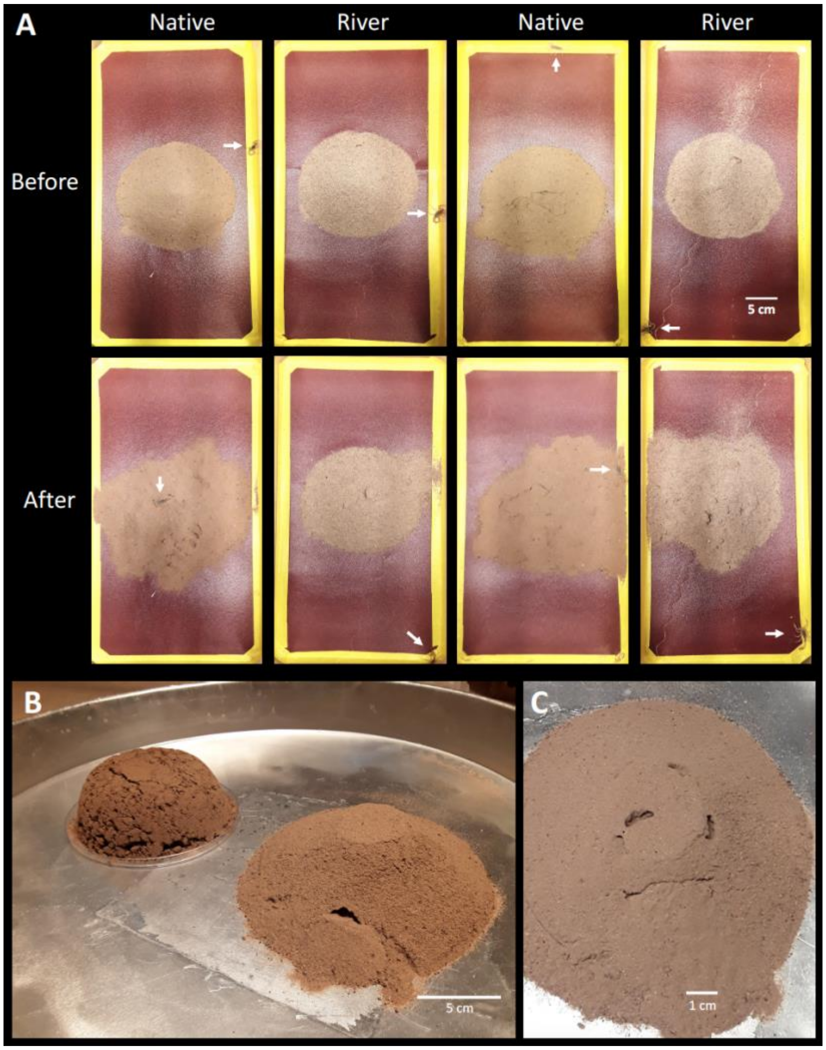

Fig. S4. Mounded substrate study. **A** Mounds of dried native substrate and river sand were formed atop sandpaper-lined boxes. Each mound was made by filling dry substrate into a Chobani yogurt cup (250 ml) and inverting using a piece of cardboard, which was slid out once the cup was placed in the middle of the box. The cup was then lifted straight up to create a well-shaped mound. Next, we misted each mound with 20 sprays of water (~15 ml). Individual male *P. utahensis* (white arrows) were introduced at 17:30 and allowed to roam through the following afternoon. Four droplights were arranged a meter above the arenas and plugged into a timer that turned the lights on at 6:00 and off at 20:00. Burrowing was pronounced in the native substrate mounds. **B,C** Pre-moistened vs dry/misted mound study. We used 500 ml of substrate and used a cereal bowl to form the mounds. We inverted the substrate in the bowl over a large Petri dish lid and did not remove the underlying Petri dish after placement in the center of the arena. The dry mound was misted from above with 40 squirts (~30 ml) of water. The pre-moistened sand was first mixed with 50 ml of water before adding to the bowl and inverting over the Petri dish lid. We used small metal drain pans (50 cm diam.) without any lining of sandpaper or substrate and set the two arenas under the four droplights on timers (described above). We placed the animals in the arenas at 17:30 and checked on them at 14:00 the following afternoon. The river sand animal made a perfect burrow near the base of the dry/misted mound and was inside (B) while the native substrate animal had not dug anything and was still out on the metal pan floor. At 14:30 we over sprayed all four mounds with five squirts of water. By the following day, the native substrate animal had made several burrows (C) in the dry/misted mound (none seen in the pre-moistened mound). The larger volume, misting from above, and the Petri dish below all appeared to be conducive to digging and helped with burrow stability.

Journal of Experimental Biology • Supplementary information

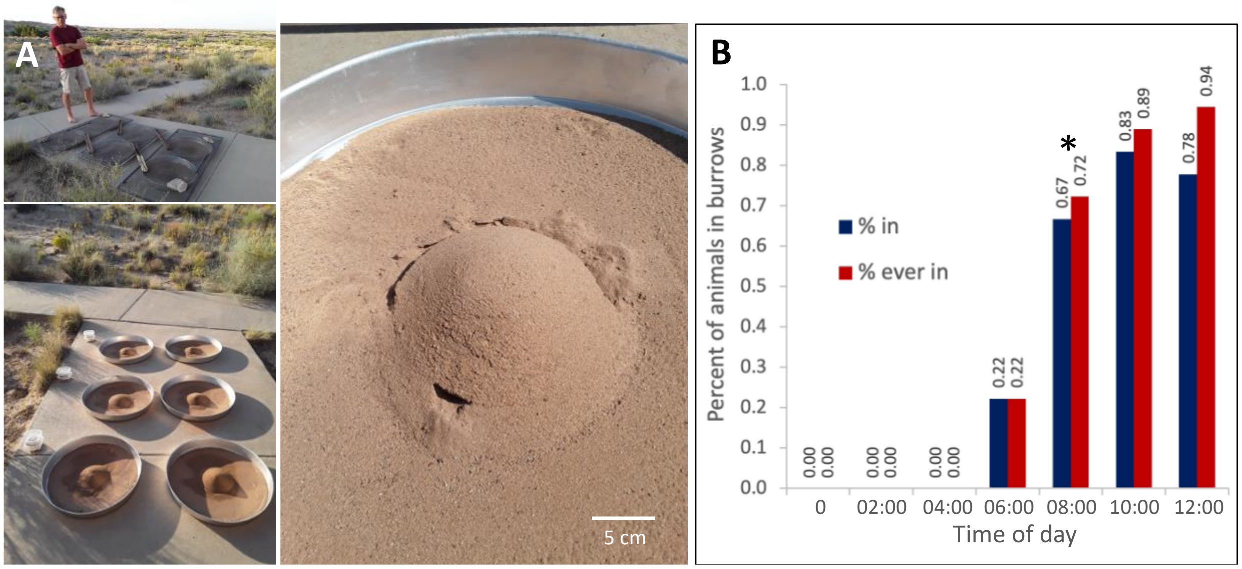

Fig. S5. Burrow occupancy study. **A** Drain pans (50 cm diam) each containing 500 ml of native substrate and a central mound formed from an additional 500 ml and over-misted with 30 ml of water are arranged on a sidewalk at the Sevilleta research station. In late afternoon (~18:00), the arenas each received a male *P. utahensis* and were covered with screens to prevent animal escape and exclude predators. The animals were monitored periodically throughout the evening. The closeup shows an example of a well-formed burrow in one of the mounds the following morning. **B** Histogram shows the number of animals inside a burrow in the central mound by time of day for the three nights of trials. Shown are both percentage of the 18 total animals either inside a burrow at the time of observation (blue bars) or that were ever in a burrow during the trials (red burrows). The asterisk indicates initial time when more than 67% of animals were inside a burrow in the mound. Across all three sets of trials, 17 of the 18 animals had occupied a burrow in the mound at some point during the observations. The animals were mostly spotted along the walls through the evening after introduction to the arena and began occupying their mounds by the following morning, ramping up precipitously between 6:00 and 8:00.

Journal of Experimental Biology • Supplementary information

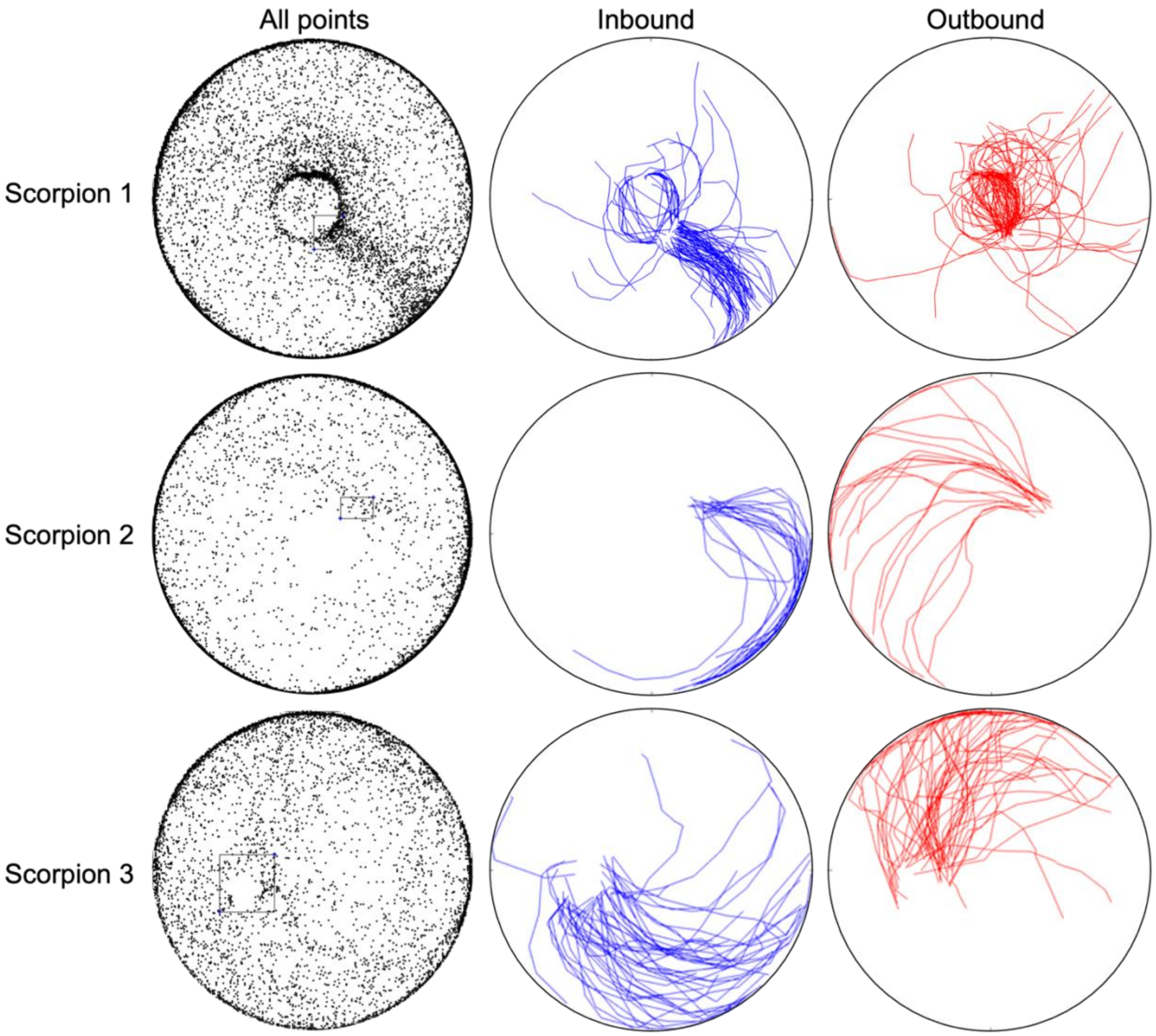

Fig. S6. Inbound vs outbound paths. Shown are some all-night plots where we drew a small rectangle around the burrow region and used MATLAB to plot the previous 20 seconds and subsequent 20 seconds of movement to that area. The animals used consistent inbound paths (blue), which were different from the outbound paths (red).

Journal of Experimental Biology • Supplementary information

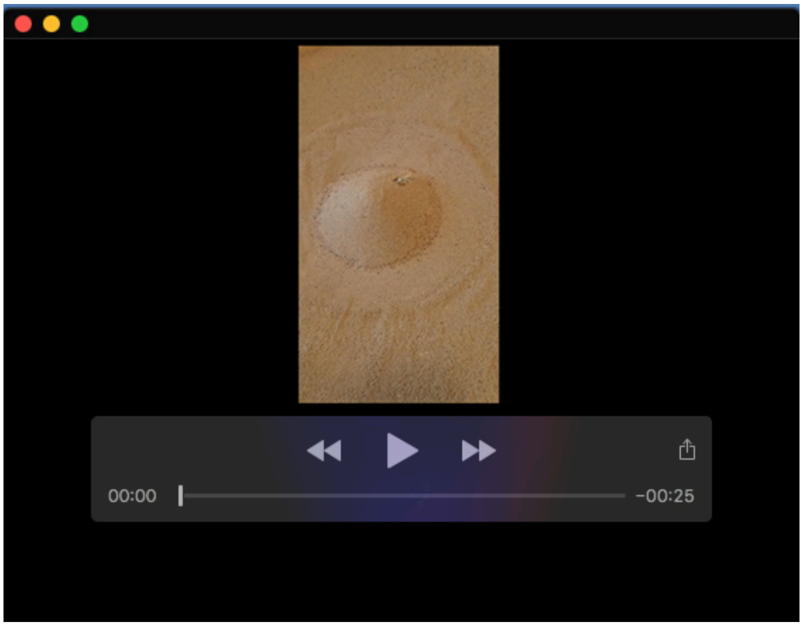

Movie 1. Sample burrow digging. This video shows an example of an animal (*P. utahensis*) digging a burrow in a laboratory arena.

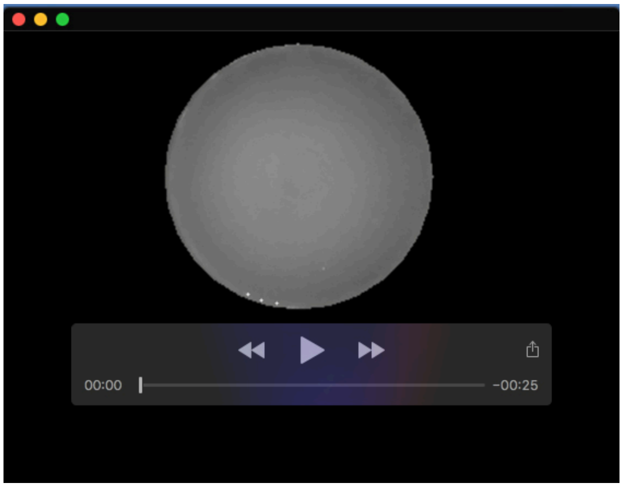

Movie 2. Sample learning walk. To find examples of putative learning walks, we first time-lapsed the all-night recordings down to short videos that we could scroll through. We wrote the MATLAB script to plot the animal location, along with the previous three frames, to create a stardust effect to better monitor the animal. We then looked for the first sign of lingering or digging in the middle of the arena and followed the subsequent movements until the animal was clearly back at the wall or remained in the burrow for a prolonged period. This clip is of a male *P. utahensis* that started digging at ~5:00 AM of a 16-hour video that started at 17:45 (5:45 PM) on Oct 26, 2020.The digging begins at ~9 s of this 21 s clip. The frame rate of the video was 0.5/s; the actual duration of this time-lapsed example was ~3.5 minutes.
